# Defect Analysis of Surface Cracks in Mn18Cr2 High-Manganese Wear-Resistant Steel Plate

**DOI:** 10.3390/ma19020241

**Published:** 2026-01-07

**Authors:** Dongjie Yang, Ning Zhang, Zhihao Liu, Bo Jiang

**Affiliations:** 1 Department of Materials and Energy, Shanxi Institute of Mechanical & Electrical Engineering, Changzhi 046011, China; ydj2005_2006@163.com; 2 School of Materials Science and Engineering, University of Science and Technology Beijing, Beijing 100083, China; 17853308824@163.com (N.Z.); 13721672615@163.com (Z.L.)

**Keywords:** Mn18Cr2, wear-resistant steel, surface crack, microstructure, Vickers hardness

## Abstract

In order to determine the causes of crack defects in Mn18Cr2 high-manganese wear-resistant steel plates, this paper conducted a systematic analysis of the steel plates’ microstructure, chemical composition, and hardness via metallographic microscopy, field-emission scanning electron microscopy, and Vickers hardness tester. The results indicated that there were folded cracks on the surface of the steel plate. The interior of the cracks was oxidized, and inclusions were observed in the crack gaps. A significant difference in the contents of Mn and Cr elements was detected at the defect locations, indicating that very obvious long-range diffusion of Mn and Cr elements had occurred during long-term high-temperature oxidation. The crack defects on the surface of the steel plate were related to the inheritance of the original cracks on the surface of the cast billet before rolling. There were cracks on the surface of the cast billet; the oxide scale and inclusions inside the cracks had not been completely removed. Multiple passes of rolling led to the cracks and oxide scale being pressed into the steel surface, thereby forming folding defects. The fine grain strengthening and deformation twinning generated by rolling deformation formed the hardened layer on the surface, resulting in higher surface hardness than core hardness. The austenite grain size inside the steel plate was in the range of 23–30 μm, and the hardness was around 275 HV. The grain size near the surface of the steel plate was around 10 μm. The surface hardness was 351 HV, which was higher than the core hardness of the steel plate.

## 1. Introduction

Wear-resistant steel plates, as key foundational materials in industrial production, are widely used in the components of large mining and construction machinery such as excavators, crushers, and ball mills [1,2,3]. They are indispensable core equipment in the fields of resource development and infrastructure construction. In service environments with continuous friction and wear, such as ore crushing, abrasive wear, and material transportation, liners serve as the core component of the abrasive wear system, bearing a crucial mission. To ensure that the liners can efficiently resist continuous impacts and abrasion from the abrasives, thereby ensuring stable and efficient operation of machinery, the performance and quality control of wear-resistant steel plates are critical. Currently, materials used in the production of wear-resistant parts include high-chromium cast iron, wear-resistant alloy steel, and high-manganese steel [4,5,6]. High-manganese steel, due to its simple production process, low production cost, and excellent mechanical properties, has been widely used as the preferred material for high-stress impact load wear conditions [7,8,9].

Mn18Cr2 high-manganese wear-resistant steel is an improvement over traditional high-manganese steel (Mn13) by increasing the content of Cr and Mn elements, thereby enhancing its water toughness treatment and deformation hardening capacity to meet the need for higher wear resistance [10,11]. After heat treatment, the microstructure of Mn18Cr2 is single-phase austenite at room temperature. Under large impact loads and contact conditions, the surface structure undergoes work hardening, transforming into martensite with high hardness, while the core maintains the ductile and tough austenitic structure [12,13]. Mn18Cr2 medium-thick plates, owing to their unique mechanical properties and material characteristics, are widely used in wear-resistant parts such as crusher mill liners, ball mill liners, and excavator bucket teeth. However, in recent years, surface defects in these medium-thick plates have become increasingly prominent, drawing significant attention within the industry [14,15]. These surface defects not only severely affect the surface quality of the plates but, more critically, they significantly degrade their mechanical properties. This reduction in load-bearing capacity poses serious risks to the reliability of large mechanical equipment and may even lead to catastrophic consequences [8].

This paper investigates the surface cracks and other defects found in 12 mm thick Mn18Cr2 steel plates produced by a certain factory. The microstructure, composition, and hardness of the material are analyzed to accurately determine the specific causes of the defects, thereby providing a solid theoretical basis for the formulation of effective improvement measures and optimization of the production process.

## 2. Experimental Materials and Methods

**Figure 1:**
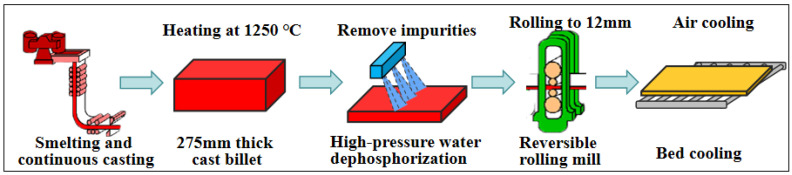
Production process of 12 mm thick Mn18Cr2 steel plate.

The experimental material consists of 12 mm thick Mn18Cr2 steel plates produced by a certain company (chemical composition: 1.03% C, 0.37% Si, 18.10% Mn, 1.95% Cr, 0.032% P, 0.001% S, 0.09% Mo, 0.034% Al, 0.04% Cu, 0.075% Ti, 0.13% V, 0.007% Nb, wt.%). The production process of the steel plates is shown in Figure 1. A 275 mm thick × 1230 mm wide casting was heated to 1250 °C in a furnace and then hot-rolled. A reversible rolling mill is used for the reciprocating rolling process, with high-pressure water dephosphorization equipment employed to remove surface oxides and other impurities before each pass. The 275 mm thick casting was subjected to 26 consecutive hot rolling passes, reducing the thickness to 12 mm without intermediate heating, and then air-cooled to room temperature on a cooling bed. During production, surface cracking defects have been observed in certain areas of the steel plates.

**Figure 2:**
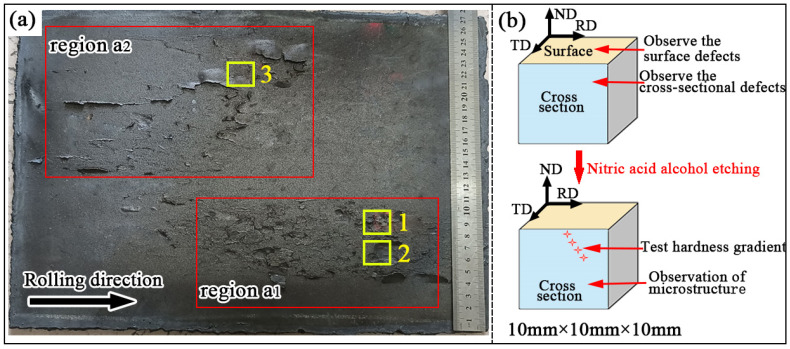
Macroscopic morphology of surface defects on Mn18Cr2 steel plate and sampling diagram (**a**) macroscopic morphology of steel plates; (**b**) sample size and characterization method. (Abbreviations: RD = Rolling Direction; TD = Transverse Direction; ND = Normal Direction).

The morphology of the steel plates is shown in Figure 2a, where surface defects are observed in certain areas. This sample was taken from the central region of the 12 mm-thick finished plate, while avoiding both the head and tail ends of the rolled plate. In region a_1_, more severe surface cracks are present, while region a_2_ also shows numerous cracks. To examine the microstructure of the samples and investigate the causes of surface defects, small samples measuring 10 mm × 10 mm × 10 mm were cut from positions 1, 2, and 3 in the defect areas of region a_1_ and region a_2_ for characterization analysis. The sampling locations and the sample characterization methods are illustrated in Figure 2b.

The samples with surface defects (1, 2, and 3) were cleaned in an ethanol solution using an ultrasonic cleaner for 3 min, then air-dried. The surface defects were observed using a field-emission scanning electron microscope (FESEM) equipped with an energy-dispersive X-ray spectrometer (EDS) for morphology observation and composition analysis. After polishing and mechanical grinding of the bulk samples 1, 2, and 3 with sandpaper, surface crack morphology in the polished state was examined using both a metallurgical microscope and FESEM. The cross-sections of all the samples were polished with sandpaper and mechanically polished, followed by etching with a 4% nitric acid-ethanol solution and electrolytic polishing. The microstructure from the edge to the center of the steel plate along the thickness direction was observed using both a metallurgical microscope and FESEM. The hardness gradient was measured using a micro Vickers hardness tester, with hardness points spaced approximately 100 μm apart along the thickness direction of the steel plate from the surface to the center. The applied load was 500 g, and the loading time was 15 s.

## 3. Results and Discussion

### 3.1. Observation and Analysis of Surface Cracks

**Figure 3:**
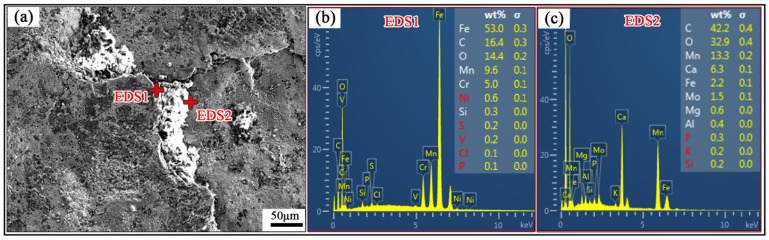
Microscopic morphology and energy spectrum of the surface crack on unpolished steel plate (**a**) macroscopic morphology of surface crack on steel plates; (**b**,**c**) energy spectrum.

Figure 3 shows the microstructure and EDS analysis of the rolled surface of the Mn18Cr2 steel plate in its unpolished state at the crack site. The micrographs in this figure correspond to sample 3 from Figure 2a. It is evident that the surface of the steel plate has long cracks, and both the cracks and their surrounding areas contain numerous impurities. EDS analysis at position 1 reveals 14.4% O and 53.0% Fe, indicating that the surface of the steel plate is fully oxidized, with the fractured area mainly consisting of the oxide layer on the rolled surface. Further analysis of the material in the center of the oxide layer cracks detected higher amounts of O, Ca, Mg, Al, and other elements. Among them, the Ca element with relatively high content was mainly derived from the calcium-aluminum deoxidizers added during steelmaking, slag, and refractory materials in the smelting furnace lining.

**Figure 4:**
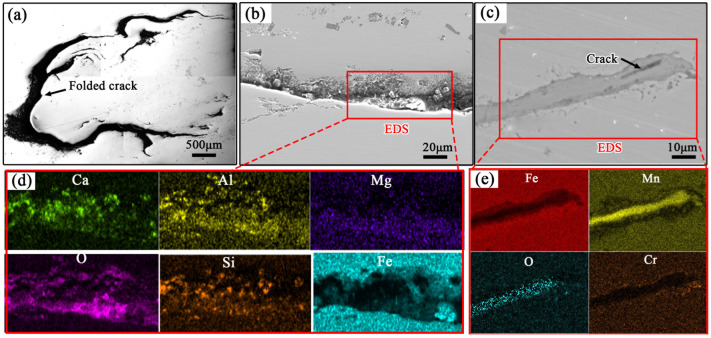
Microscopic morphology and energy spectrum of the surface crack on polished steel plate (**a**,**b**) morphology of surface cracks; (**c**) cross-sectional morphology of cracks; (**d**,**e**) energy spectrum.

To investigate whether there are any inclusions inside the cracks, the polished surface morphology of the defective steel plate sample was observed using FESEM, and EDS analysis was performed, as shown in Figure 4. The crack defects are observed to exhibit a folding pattern in Figure 4a. Numerous studies have shown that folding defects are primarily formed during the rolling process [16,17,18]. If there are cracks on the surface of the continuous cast billet or inclusions beneath the surface of the steel billet, these cracks may be rolled into the steel plate during subsequent rolling, forming folds. Uneven metal flow around the inclusions can also easily cause folding on the metal surface [19,20]. Additionally, improper rolling processes and issues such as edge defects or burrs in the billets can also lead to the formation of folding crack patterns [21,22]. Figure 4b shows the polished surface crack morphology of the rolled steel plate and the corresponding EDS surface scan analysis. The analysis detected significant amounts of Ca, Al, O, Mg, and other elements in the crack material. Moreover, a large amount of bright white material was found in the center of the crack, which also contained Ca, Al, and other elements. This suggests that the crack inside the steel plate has oxidized, and the gaps in the surface cracks contain many oxides and inclusions. FESEM observation of the cross-sectional crack morphology and EDS analysis are shown in Figure 4c, where an accumulation of Mn and O elements is observed around the crack. Due to selective oxidation during the heating process, there is a significant difference in the Mn and Cr content in the defective area and its vicinity, indicating that during prolonged high-temperature oxidation, Mn and Cr elements exhibit notable long-range diffusion. This differs greatly from the incomplete oxidation and short-range diffusion of the rolling cracks [23]. Based on these analysis results, it can be inferred that the surface crack defects of the steel plate were not formed during the rolling process but are related to the inherited original cracks on the surface of the steel ingot before rolling.

### 3.2. Microstructure and Hardness Analysis

**Figure 5:**
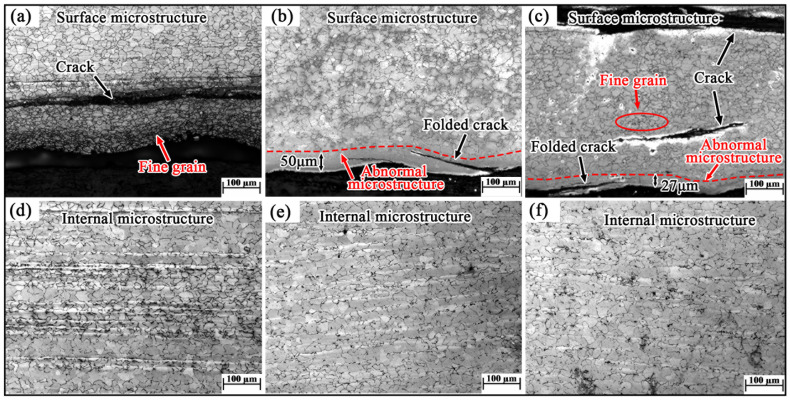
Microstructure of defect position of Mn18Cr2 steel plate (**a**,**d**) sample 1; (**b**,**e**) sample 2; (**c**,**f**) sample 3; (**a**–**c**) surface microstructure; (**d**–**f**) internal microstructure.

Figure 5 shows the optical microstructure of the cross-section of the defect area in the Mn18Cr2 steel plate. The microstructure across all regions is austenitic. The grain structure at the center is uniformly distributed, with a size range of 23–30 μm. At the surface, distinct cracks are observed in the microstructure, with some cracks exhibiting a folding pattern. Significant differences in grain size are observed on either side of the cracks. As shown in Figure 5a, the grain size inside the crack is coarser, approximately 25 μm, which is similar to the grain size of the normal microstructure. The grain size near the surface of the crack is relatively finer, with an average size around 10 μm. Additionally, some areas with cracks exhibit an “abnormal microstructure” similar to decarburized layers on the surface, where no grain boundaries are observed, as shown in Figure 5b,c.

**Figure 6:**
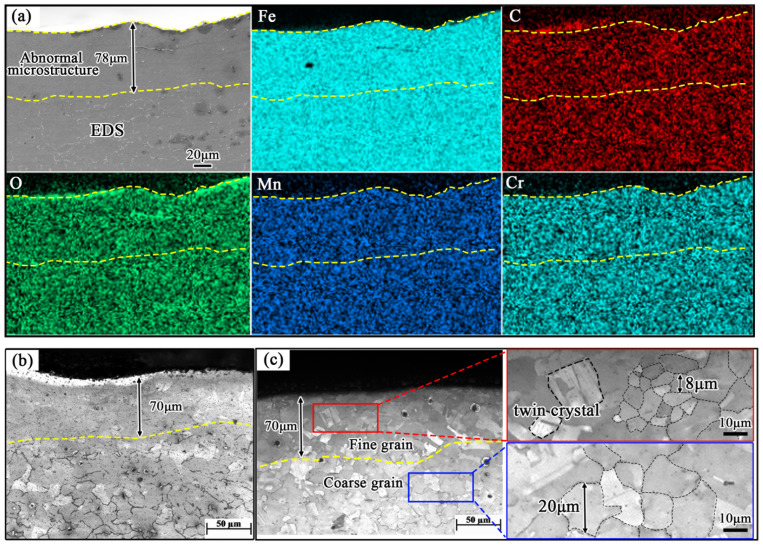
Surface energy spectrum and morphology of “abnormal structure” on the defect position of Mn18Cr2 steel plate (**a**) surface energy spectrum; (**b**,**c**) morphology of “abnormal structure”.

To determine the microstructure type of the “abnormal microstructure” resembling a decarburized layer, sample 3 was selected for EDS surface scanning composition analysis. Figure 6a shows the surface scan results of the “abnormal microstructure.” The composition of the “abnormal microstructure” is consistent with that of the steel plate matrix, indicating that it is not a decarburized layer. Electrolytic etching was applied to the “abnormal microstructure” on the surface, using a 10% perchloric ethanol solution as the electrolyte. The resulting etched microstructure is shown in Figure 6c. When compared with the matrix microstructure, the “abnormal microstructure” exhibits significantly finer grains and a small amount of twin crystal structures.

**Figure 7:**
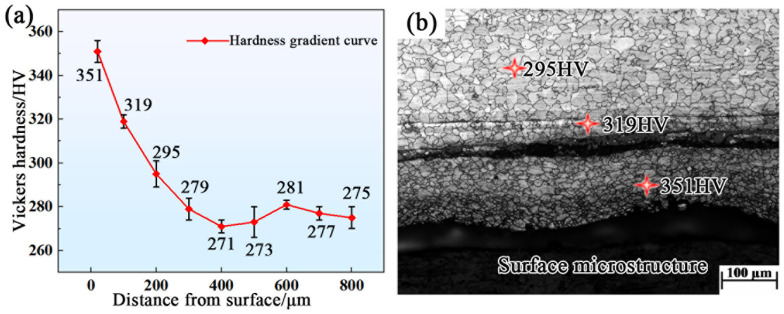
Vickers hardness gradient curves and hardness test point morphology of different position of Mn18Cr2 steel plate (**a**) hardness gradient curves; (**b**) microstructure morphology corresponding to hardness points.

The Vickers hardness tester was used to measure the hardness values of different regions of sample 1 in the defect area of the steel plate, from the surface to the center. The hardness values and their gradient variation curves are shown in Figure 7a. From the hardness gradient variation curves of different regions of the steel plate, it can be seen that the hardness decreases gradually from the surface to the center, reaching a minimum of 271 HV at 400 μm from the surface. It then slightly increases to 281 HV at 600 μm from the surface and decreases again to 275 HV at 800 μm from the surface. The hardness tends to stabilize at 300 μm from the surface, ranging between 271 HV and 281 HV. Figure 7b shows the corresponding microstructure of the hardness test points. The grain structure near the surface, on the outside of the crack, is the finest, with a hardness of 351 HV. As we move inward, the grain size increases gradually, and the hardness decreases correspondingly. It is evident that the fine grain structure and the formation of a small amount of twin crystals near the edge of the plate lead to an increase in surface hardness. The grain size at the surface of the steel plate is uneven, causing significant fluctuations in surface hardness. The uneven grain size is related to non-uniform deformation during the rolling process. In the rolling process, areas with larger deformation led to noticeable refinement of the grain size in those regions, as well as a marked increase in hardness [24,25]. Based on the above results, it can be concluded that the “abnormal microstructure” on the surface is not a decarburized layer, but rather a hardened layer with fine-grained structure. Due to the extremely fine grain size, the grain boundaries are difficult to etch, which gives the appearance of a “decarburized layer” [26].

### 3.3. Surface Defect Formation Analysis

**Figure 8:**
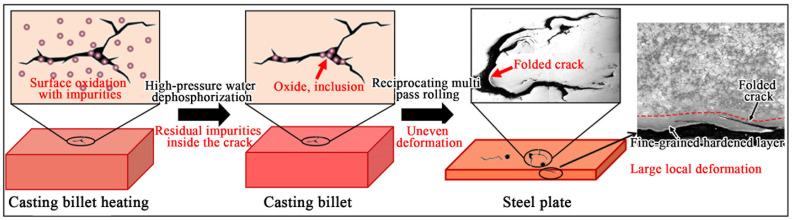
Formation mechanism of surface defects on Mn18Cr2 steel plate.

Based on the above analysis, it can be concluded that the surface crack defects of the steel plates produced by the enterprise are related to the inherited original cracks on the surface of the billet prior to rolling. Since the original casting billet used in this study was completely consumed during the rolling process, it was impossible to conduct defect characterization of the original casting billet. The core focus of this research was on the evolution mechanism of cracks during the hot rolling process, which demonstrates the importance of strictly controlling billet quality to prevent the inheritance of initial defects into the finished steel plates. Figure 8 illustrates the defect formation mechanism for the Mn18Cr2 steel plate surface. The billet already had cracks on its surface before rolling. After being heated to high temperatures, both the surface and the interior of the cracks were oxidized. Prior to each pass in the rolling process, the billet was subjected to high-pressure water dephosphorization, but the oxide scale and various oxide inclusions in the crack gaps were not entirely removed. After several passes of rolling, surface cracks and oxide scale were rolled into the steel plate, resulting in folding defects. Furthermore, the uneven metal flow around the inclusions in the crack gaps exacerbated the formation of surface folding defects. Additionally, in comparison with the 12 mm-thick plate after rolling, the 275 mm-thick casting billet features a smaller length and width as well as a lower width-to-thickness ratio. At the initial stage of rolling, the low width-to-thickness ratio of the billet exacerbated the deformation inhomogeneity, while the relatively short length compressed the steady-state rolling window in a temporal dimension. The combined effect of these two factors resulted in rolling instability and non-uniform deformation of the billet. The presence of surface defects further exacerbated this non-uniform deformation. Severe deformation of the surface resulted in the refinement of the grain structure and the formation of a small amount of twinning. The fine grains and deformation-induced twinning hindered dislocation movement, thereby enhancing the surface hardness [27,28]. This led to the formation of a hardened layer with a certain thickness on the surface, which was harder than the matrix structure [29,30]. However, due to the uneven deformation, the grain size at different parts of the surface varied, leading to significant fluctuations in surface hardness.

To avoid the formation of such surface defects, strict control of billet quality is essential to prevent the inheritance of initial defects into the finished steel plates. By providing feedback to the steelmaking plant regarding quality issues, the steelmaking process was optimized, which helped prevent the generation of surface cracks.

## 4. Conclusions

(1) The surface of the Mn18Cr2 steel plate was covered with an oxide layer, and there were crack defects in a folding form. The cracks were oxidized inside, and the crack gaps contained inclusions. Due to selective oxidation during heating, there was a significant difference in the Mn and Cr element concentrations at the defect and its surrounding areas, indicating noticeable long-range diffusion of Mn and Cr during prolonged high-temperature oxidation. The surface crack defects of the steel plate were related to the inherited original cracks on the surface of the billet prior to rolling.

(2) The microstructure of the Mn18Cr2 steel plate in all regions was austenitic. In the normal areas, the grains were uniformly distributed, with sizes ranging from 23 to 30 μm. In the defect areas of the steel plate, a fine-grained layer was observed on the outside of the crack, with an average grain size of around 10 μm. The hardness of the steel plate decreased gradually from the surface to the center, eventually stabilizing at approximately 275 HV. The grain structure near the surface outside the crack was the finest, with a hardness of 351 HV. As the depth increased, the grain size gradually grew, and the hardness decreased correspondingly. The uneven grain size was related to the uneven deformation during the rolling process. In areas with more intense deformation, the grain size was refined, and a small amount of twinning was formed, which led to an increase in surface hardness.

(3) The surface crack defects of the Mn18Cr2 steel plate were related to the inherited original cracks on the surface of the billet prior to rolling. During the high-temperature heating process, both the surface and the interior of the cracks were oxidized. Although high-pressure water dephosphorization was applied, the oxide scale and inclusions inside the cracks were not completely removed. Multiple passes of rolling caused the surface cracks and oxide scale to be rolled into the steel plate, forming folding defects. The dimensional characteristics of the billet led to instability and uneven deformation during rolling, which further exacerbated the uneven deformation caused by the surface defects. The fine grain strengthening and deformation-induced twinning produced by severe surface deformation led to the formation of a hardened layer, with the surface hardness being higher than that of the center. Therefore, strict control of billet quality is necessary to prevent the inheritance of original defects into the finished steel plates.

## Data Availability

The original contributions presented in this study are included in the article. Further inquiries can be directed to the corresponding author.
